# Estimating sensitivity and specificity of diagnostic tests using latent class models that account for conditional dependence between tests: a simulation study

**DOI:** 10.1186/s12874-023-01873-0

**Published:** 2023-03-10

**Authors:** Suzanne H. Keddie, Oliver Baerenbold, Ruth H. Keogh, John Bradley

**Affiliations:** 1 Department of Infectious Disease Epidemiology, London School of Hygiene and Tropical Medicine, London, UK; 2 Department of Medical Statistics, London School of Hygiene and Tropical Medicine, London, UK; 3 MRC International Statistics and Epidemiology Group, London School of Hygiene and Tropical Medicine, London, UK

**Keywords:** Diagnostic test accuracy, Latent class model, Bayesian inference, Conditional dependence

## Abstract

**Background:**

Latent class models are increasingly used to estimate the sensitivity and specificity of diagnostic tests in the absence of a gold standard, and are commonly fitted using Bayesian methods. These models allow us to account for ‘conditional dependence’ between two or more diagnostic tests, meaning that the results from tests are correlated even after conditioning on the person’s true disease status. The challenge is that it is not always clear to researchers whether conditional dependence exists between tests and whether it exists in all or just some latent classes. Despite the increasingly widespread use of latent class models to estimate diagnostic test accuracy, the impact of the conditional dependence structure chosen on the estimates of sensitivity and specificity remains poorly investigated.

**Methods:**

A simulation study and a reanalysis of a published case study are used to highlight the impact of the conditional dependence structure chosen on estimates of sensitivity and specificity. We describe and implement three latent class random-effect models with differing conditional dependence structures, as well as a conditional independence model and a model that assumes perfect test accuracy. We assess the bias and coverage of each model in estimating sensitivity and specificity across different data generating mechanisms.

**Results:**

The findings highlight that assuming conditional independence between tests within a latent class, where conditional dependence exists, results in biased estimates of sensitivity and specificity and poor coverage. The simulations also reiterate the substantial bias in estimates of sensitivity and specificity when incorrectly assuming a reference test is perfect. The motivating example of tests for Melioidosis highlights these biases in practice with important differences found in estimated test accuracy under different model choices.

**Conclusions:**

We have illustrated that misspecification of the conditional dependence structure leads to biased estimates of sensitivity and specificity when there is a correlation between tests. Due to the minimal loss in precision seen by using a more general model, we recommend accounting for conditional dependence even if researchers are unsure of its presence or it is only expected at minimal levels.

**Supplementary Information:**

The online version contains supplementary material available at 10.1186/s12874-023-01873-0.

## Background

Diagnostic tests are used widely to discriminate between individuals with and without certain conditions and diseases. The results of these tests have important consequences, for both decision on treatment of individuals and for population health interventions. As a result, accurate characterization of diagnostic tests is paramount for optimal decision making. The usefulness of a diagnostic test is a combination of its accuracy, namely sensitivity and specificity, as well as practical considerations including cost, ease of use and speed of results. Because of this variety of factors to consider, which can involve difficult tradeoffs, new tests are continually being developed that aim to improve upon previous tests in any of these factors. To truly compare test effectiveness we must be able to assess the accuracy of a diagnostic test with minimal bias and high precision.

Standard methods estimate the sensitivity and specificity of a diagnostic test by comparing the results of a new test to the results of a ‘gold standard’ reference test. On the assumption that the reference test is indeed a ‘gold-standard’ or perfect test, with 100% sensitivity and 100% specificity, we can be certain of the true infection status of each individual tested and we can estimate the sensitivity and specificity of the new test directly. However, diagnostic tests are rarely perfect and in some instances there is no gold-standard test with which to compare. Examples of pathogens and infections where this is the case include Tuberculosis [1], Schistosomiasis [2] and Influenza [3]. In this situation, estimating the sensitivity and specificity of a diagnostic test is a difficult statistical problem and naively assuming the reference test is perfect will result in biased estimates of the new test’s accuracy [4]. However, the accuracy of a given test can still be estimated by comparing the results of multiple imperfect tests applied to the same group of people. An increasingly popular method for making use of data on results from multiple imperfect diagnostic tests uses Bayesian latent class models (LCM) [5] and this approach has been applied across a wide range of pathogens [6–8].

To estimate diagnostic test accuracy with data from multiple imperfect tests using LCM requires making assumptions. Simple LCMs make the assumption that, conditional on the true infection status, results from multiple tests on an individual are independent. That is, the result of one test provides no information about the result of another test given the infection status of an individual. We refer to this situation as *conditional independence* throughout the rest of this paper. It has been highlighted by several researchers [9, 10] that the conditional independence assumption is unlikely to hold. For example, the assumption is unlikely to hold when there is a spectrum of disease severity. It is likely to be easier to detect disease in more severe cases for many pathogens and therefore, different tests on the same individual are more likely to return the same result. When disease severity, or some other factor associated with an individual, is associated with ease of detection, there remains a dependence between the tests even after conditioning for the true infection status of an individual. Tests that are based on the same underlying mechanism are also unlikely to be independent given the individual’s disease status. When the assumption of conditional independence between tests is not valid, an analysis that assumes such independence is expected to result in biased estimates of sensitivity and specificity [11]. The assumption of conditional independence can be relaxed through incorporating either fixed [9] or random effects [12] into the LCM. The implementation of both in a Bayesian framework has been described elsewhere [11].

In a simulation study by Wang et al. [13] the authors showed that LCM with fixed effects, to account for conditional dependence among disease positive individuals, worked well both when tests were highly correlated (conditionally dependent) and when tests were truly conditionally independent. They also showed that the use of fixed effects or random effects has very little impact on the overall estimates of test accuracy. However, they only explored the possibility of conditional dependence in disease positive individuals as they assumed all diagnostic tests had a specificity of 99%. As a result, there could be no, or negligible, conditional dependence between these tests among disease negative individuals. However, the assumption of 99% specificity may not hold in many cases, so conditional dependence in non-infected individuals is also a possibility. When this is the case, researchers have a choice of conditional dependence structure in infected or non-infected individuals or both, and should be aware of the impact on estimates of sensitivity and specificity from choosing a particular structure, a situation highlighted in the case study by Menten et al. [14].

Much of the literature to date has focused on the importance of accounting for conditional dependence in disease positive individuals with much less discussion on the importance of conditional dependence among disease negative individuals. Above we discuss that disease severity or intensity may explain conditional dependence in disease positive individuals, In disease negative individuals, the presence of other parasites may work in a similar way, leading to a higher probability of false positive results on a range of tests, thus inducing a positive correlation among test results and a dependence between test results conditional on the true infection status [15].

There has been little research on the impact on estimates of sensitivity and specificity of choosing to account for conditional dependence in disease positive or disease negative individuals only versus accounting for conditional dependence in both. Here we focus on LCM with random effects and carry out a simulation study investigating the size of bias and impact on precision of estimates of sensitivity and specificity and coverage of 95% credible intervals, when the conditional dependence structure is mis-specified. We also look at how the size of this bias changes depending on the level of dependence between tests. Finally, we extend the analysis from a motivating example that estimated the accuracy of five different diagnostic tests used in the identification of Melioidosis [16], to highlight the importance of the conditional dependence structure chosen in practice.

## Methods

We begin this section with an overview of the latent class models used for diagnostic test accuracy before introducing our motivating example, followed by details of the simulation study utilizing the structured approach developed by Morris et al. [17].

### Latent class models

We consider a sample of *N* individuals who all undergo *R* binary diagnostic tests. We have observed data ***Y =*** {***y***_***ij***_; ***i =*** **1**, …, ***N***, ***j =*** **1**, …, ***R***} where *y*_*ij*_ represents the test result (1 = positive, 0 = negative) of the *j*th test for the *i*th individual. We assume two disease classes, and we let *d*_*i*_ denote the true (but unobserved) infection status for individual *i*, with those who are truly infected having *d*_*i*_ = 1 and those who are truly not infected *d*_*i*_ = 0. The disease prevalence (i.e. the proportion for whom *d*_*i*_ = 1) in the underlying population is denoted *π*.

For a given test, the probability that an individual who is truly infected will return a positive test result is defined as the sensitivity (*Se* = Pr(*y* = 1| *d* = 1)) and the probability that an individual who is truly not infected will return a negative test result is defined as the specificity (*Sp* = Pr(*y* = 0| *d* = 0)). Each test *j* has its own sensitivity and specificity, denoted *Se*_*j*_ and *Sp*_*j*_. Under the assumption that the *R* diagnostic tests are conditionally independent, the likelihood of the observed data can be expressed as:

1 $$\textit{P}\mathit{\left({\boldsymbol Y\;\vert\;\pi,Se,Sp}\right)}\mathit=\overset{\mathit N}{\underset{\mathit i\mathit=\mathit1}{\mathit\prod}}\mathit{\left({\left(\pi\overset R{\underset{j=1}{\mathrm\prod}}{Se_j}^{y_{ij}}\left(1-{Se}_j\right)^{1-y_{ij}}\right)+\left(\left(1-\pi\right)\overset R{\underset{j=1}{\mathrm\prod}}{Sp_j}^{\left(1-y_{ij}\right)}\left(1-{Sp}_j\right)^{y_{ij}}\right)}\right)}$$

To account for conditional dependence between tests in either or both truly infected individuals or truly not infected individuals, we allow the sensitivity and/or the specificity to vary by individual using a random effect. This reflects the situation where some subject-specific characteristic, besides the true disease status of the individual, affects the test result seen. The subject-specific value of the *i*^*th*^ individual in a disease class is denoted by *s*_*id*_. Then, we can define the sensitivity of the *j* th test for the *i* th individual as *Se*_*ij*_ = Pr(*y*_*ij*_ = 1 ∣ *d*_*i*_ = 1, *s*_*i*1_) and similarly the specificity as *Sp*_*ij*_ = Pr(*y*_*ij*_ = 0 ∣ *d*_*i*_ = 0, *s*_*i*0_). The likelihood in [1] is then modified to include *Se*_*ij*_ and *Sp*_*ij*_ where we assume then that sensitivity takes the form:

2 $${Se}_{ij}={\textrm{g}}^{-1}\left({\alpha}_{\textrm{j}1}+{\upbeta}_{\textrm{j}1}{s}_{\textrm{i}1}\right)$$

and, specificity:

3 $${Sp}_{ij}={\textrm{g}}^{-1}\left({\alpha}_{\textrm{j}0}+{\upbeta}_{\textrm{j}0}{s}_{\textrm{i}0}\right)$$

where *g*(·) is a link function. In this study we use the inverse logit link, so *g*^−1^(*x*) = 1/(1 + *e*^−*x*^). *α*_*jd*_ and *β*_*jd*_ are unknown parameters to be estimated. *β*_*jd*_ describes the dependency of test *j* in disease class *d* on the random effects such that if all *β*_*jd*_ = 0, there is no dependence on the random effect and all *j* tests among both disease classes are conditionally independent. We can estimate the mean or median sensitivity and specificity of a given test from the two parameters *α*_*jd*_ and *β*_*jd*_. The random effect *s*_*id*_ is assumed to follow a standard normal distribution (*s*_*id*_~*N*(0, 1)) . For a more detailed description of random-effect latent class models see references [11, 12] and for details about how latent class models are implemented in this study see the model specification and implementation section below.

### Motivating example

We illustrate the impact of different conditional dependence structures on estimates of sensitivity and specificity using data from a study that utilised LCM to estimate the sensitivity and specificity of five different diagnostic tests used in the diagnosis of Melioidosis [16]. Melioidosis is an infectious disease caused by the bacterium *Burkholderia pseudomallei*. The data are from a cohort of 320 febrile adult patients recruited over a 6 month period from a hospital in the northeast of Thailand in 2004 [18]. The five tests included four serological tests (indirect hemagglutination test (IHA), IgM immunochromogenic cassette test (ICT), IgG ICT, and ELISA) and culture test which was assumed 100% specific throughout all their analyses. For comparability we made the same assumption.

In the original analysis, Limmathurotsakul et al. implemented four different LCM with various conditional dependence structures as well as an analysis which assumed culture was a perfect gold standard. The LCM models varied from a model assuming conditional independence between all tests (Model 0) to those considering conditional dependence between a single pair of serological tests using fixed effects (Models 1 and 2) and finally those that use random effects to represent dependence between all serological tests within a disease class (Models 3 and 4) but they did not consider a model that simultaneously accounted for conditional dependence within both true positive and true negative individuals. See Table 1 for a summary of the models considered in the original paper. We extend their analysis to consider a ‘Model 5’ which allows dependence between all four serological tests among those individuals truly infected and those individuals truly not infected using random effects. Before reporting the results of this analysis we describe a simulation study used to explore the impact on estimates of sensitivity and specificity of using the wrong conditional dependence structure.

**Table 1:** Models and conditional dependence structures compared

Model	Dependence Structure	Effect Type Used	Included in this paper’s simulation
**Model 0**	Conditional Independence between all tests	NA	Yes
**Model 1**	Dependence between IHA and IgM ICT in disease positive individuals	Fixed	No
**Model 2**	Dependence between IHA and IgG ICT in disease positive individuals	Fixed	No
**Model 3**	Dependence between all serological tests in disease positive individuals	Random	Yes
**Model 4**	Dependence between all serological tests in disease negative individuals	Random	Yes
**MODEL 5**	Dependence between all serological tests in disease positive and disease negative individuals	Random	Yes

Models 0–4 considered in Limmathurotsakul et al. [14]. Model 5 an extension not considered in the previous analyses. The last column highlights the scenarios that are considered in the simulation in this paper

### Simulation study

#### Aim

To evaluate the impact of mis-specifying the conditional dependence structure in latent class analysis on bias, coverage, and precision of estimates of sensitivity and specificity.

### Data generating mechanism

Data are simulated on 500 individuals for five diagnostic tests. As in our motivating example, we imagine four tests (*j* = 2, 3, 4, 5), of a similar nature to the serological tests in the motivating example, that exhibit different conditional dependence structures among themselves, and one test (*j* = 1), of a similar nature to a culture test, which is assumed independent of the serological tests. We consider four scenarios for the conditional dependence structure between serological tests described in Table 2. In all four conditional dependence scenarios, the underlying disease prevalence is 50% (*π* = 0.5). All tests have a median sensitivity of 0.65 (*g*^−1^(*α*_1*j*_) = 0.65) while the median specificity of the four serological type tests is 0.9 (*g*^−1^(*α*_0*j*_) = 0.9, *j* = 2, .., 5) and the median specificity of our independent reference culture type test is 0.99 (*g*^−1^(*α*_01_) = 0.99).

**Table 2:** Data generating mechanisms considered

Data Generating Mechanism	Dependence in disease positive Individuals	Dependence in disease negative Individuals	Value of ***β***_***jd***_ in models (2) and (3) for sensitivity and specificity
**CIndep**	No	No	*β*_*jd*_ = 0, *d* = 0, 1
**CDP**	Yes	No	*β*_*j*1_ = 1, *β*_*j*0_ = 0
**CDN**	No	Yes	*β*_*j*0_ = 1, *β*_*j*1_ = 0
**CDPN**	Yes	Yes	*β*_*jd*_ = 1, *d* = 0, 1

For the three scenarios in which there is conditional dependence, we set *β*_*jd*_ equal to 1. When the median sensitivity is 65%, the inclusion of this random effect means the interquartile range for sensitivity is 48–78% and with a median specificity of 90% the interquartile range is 82–94%. In a secondary simulation, we also compared this scenario with two additional scenarios under different values for *β* (*β* = 0.2,0.6), where lower values of the standard deviation correspond to a narrower interquartile range around the median sensitivity and specificity.

### Estimand/target of the simulation

In each simulated data set we estimate the sensitivity and specificity of each diagnostic test (*j* = 1, .., 5) for the median individual (with random effect 0) and the associated 95% credible interval.

## Methods

Each simulated dataset is analysed in the following five ways:

1. **GS Model:** A conditionally independent model where test 1 (culture) is assumed perfect, i.e. a gold standard model (GS) (*Se = Sp = 1*)
2. **CIndep Model:** A conditionally independent (CIndep) model where test 1 (culture) is assumed imperfect (Eq. 1)
3. **CDP Model:** A model allowing conditional dependence in disease positive (CDP) individuals only (among serological tests, *j* = 2, . . , 5) and all tests (*j* = 1, .., 5) are assumed imperfect (Eq. 2)
4. **CDN Model:** A model allowing conditional dependence in disease negative (CDN) individuals only (among serological tests, *j* = 2, . . , 5) and all tests (*j* = 1, .., 5) are assumed imperfect (Eq. 3)
5. **CDPN Model:** A model allowing conditional dependence in both disease positive and disease negative (CDPN) individuals (among serological tests, *j* = 2, . . , 5) and all tests (*j* = 1, .., 5) are assumed imperfect (Eqs. 2 and 3)

### Performance measures

Under each scenario, we generated 1000 simulated data sets. We assess performance through bias in estimates of sensitivity and specificity (including the Monte Carlo standard errors), precision of those estimates measured by the empirical standard error, and the coverage of the 95% credible intervals. These measures are defined in Supplementary Table 1. Empirical diagnostics were recorded for all simulations to keep track of any simulations with inference validity concerns. Validity concerns occurred when either divergent transitions and/or the split $\hat{R}$ statistic values larger than 1.01 were recorded [19, 20]. Any simulations with validity concerns are removed from the presentation of results.

### Model specification and implementation

All models are fitted using Bayesian methods and so prior distributions must be specified for all parameters. In all models, the prior distribution for prevalence is assumed uniform between 0 and 1. In models where culture is allowed to be imperfect (CIndep, CDP, CDN and CDPN Models) the sensitivity of all tests are assumed uniform between a lower limit of 1 minus the specificity Se_jlower_ = 1 − Sp_j_, *j* = 1, . . , 5, and 1. This ensures that the probability of a positive test is higher for somebody with disease than without. In these same models, the specificity of our independent test (*j* = 1) is assumed to follow a *beta*(10, 1) prior distribution and the specificity of all other tests (*j* = 2, .., 5) is assumed to follow a *beta*(5, 1) prior distribution. Although in this simulation we are assuming test 1 is a culture test and therefore we could assume a much stronger prior distribution for specificity, for the purposes of a more generalizable simulation we have kept this relatively uninformative. Assuming a *beta*(10, 1) distribution corresponds to an assumption of 95% probability of the specificity being above 74% and a *beta*(5, 1) distribution corresponds to an assumption of 95% probability of the specificity being above 55%. Where we account for conditional dependence between tests using random effects, *β*_*jd*_ is assumed to follow a *gamma*(1, 1) prior distribution. In this paper we assume that *β* is the same between all serological type tests (*j* = 2, .., 5) but that that the culture type test (*j* = 1) is independent, and for simplicity, we consider the case where *β*_*jd*_ = *β*_*d*_, *j* = 2, . . , 5. The effect of this is that a change in the random effect *s*_*i*_ will cause the sensitivity and of all serological type tests for the *i*^*th*^ individual to change by the same amount and similarly, the specificity of all serological type tests for the *i*^*th*^ individual to change by the same amount. We implement all models in R [21, 22] using stan [23] and all code can be found at: https://github.com/shk313/Evaluating-sensitivity-and-specificity-from-LCM-a-simulation-study.git.

## Results

### Simulation study

#### Bias

**Fig. 1:**
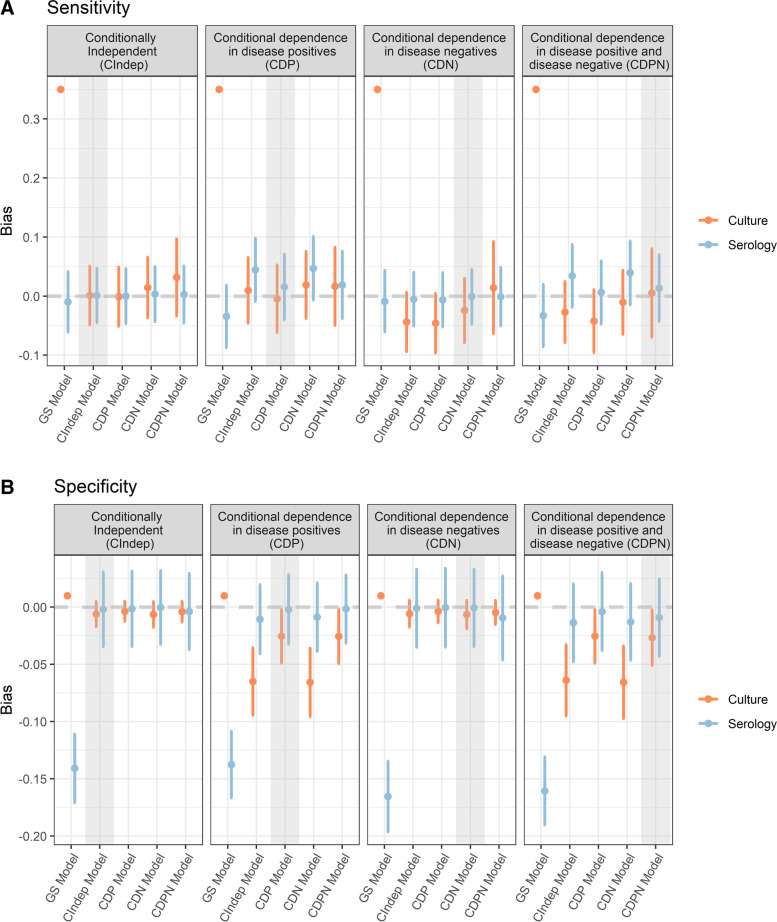
Mean bias and 95% confidence interval in estimates of sensitivity and specificity under each scenario. Points show mean bias across all valid simulations and the bar extends to the lower and upper confidence intervals. Shaded areas highlight the model that corresponds to the data generating mechanism

Throughout the presentation of the results tests 2–5 (*j* = 2, .., 5) are combined. Figure 1 shows the overall mean bias and associated 95% confidence interval which quantifies the uncertainty in the estimates of bias for median sensitivity and median specificity across all simulations (excluding those where either divergent transitions and/or the split $\hat{R}$ statistic values larger than 1.01 were recorded) for each model under each data generating mechanism. For all data generating mechanisms, use of the GS Model where test 1 (culture) is assumed perfect yields biased estimates. The sensitivity of test 1 (culture) is biased upwards because the test does not have a sensitivity of 100% as is assumed in the model and the specificity of the serological tests is underestimated by a minimum of 10% considering the upper limit of the 95% confidence interval and a maximum of 20% using the lower limit of the 95% confidence interval across all data generating mechanisms. Under the CIndep data generating scenario all other models provide approximately unbiased estimates of sensitivity and specificity, with 0 being contained within all the 95% confidence intervals.

Considering the three data generating mechanisms where there is conditional dependence among serological tests within either or both disease positive and disease negative individuals, sensitivity estimates are approximately unbiased from all models with the exception of the GS Model. Each data generating mechanism and model combination for sensitivity estimates reported a Monte Carlo standard error less than 0.02 and 6 out of 40 scenarios had a Monte Carlo 95% confidence interval that contained zero while in the remaining scenarios the confidence interval did not contain 0, suggesting there is some small bias even after accounting for sampling variability. Bias in specificity estimates among data generating mechanisms with conditional dependence is minimised when the ‘correct’ model is used. This is most notable when there is conditional dependence between tests among positive individuals. For this data generating mechanism (CDP), when the model used assumes conditional independence between tests (CIndep Model) or conditional dependence between tests in negative individuals only (CDN Model), culture specificity is underestimated but serological tests specificity estimates are approximately unbiased. For specificity all scenarios had a Monte Carlo standard error less than 0.01 and, like with sensitivity, only six scenarios had a 95% Monte Carlo confidence interval for the estimate that contained zero.

### Coverage

Tables 3 and 4 show the coverage probability, that is, the percentage of simulations where the 95% credible interval for the estimate (sensitivity or specificity) contains the true value. In the GS model where the sensitivity and specificity of culture is assumed perfect the 95% credible intervals for both estimates results in 0% coverage for the true sensitivity and specificity of culture and also 0% coverage for the specificity of serological tests across all data generating mechanisms. For sensitivity, all models except the GS Model show good coverage under the CIndep scenario. With conditional dependence among serology tests in disease positive individuals, models which do not account for this dependence have coverage around 80% for serology tests (*j* = 2, .., 5). On the other hand, when there exists conditional dependence between tests *j* = 2, . . , 5 in disease negative individuals only, the coverage in those models that do not account for dependence remains close to 95% for serology tests but is below 85% for culture. The coverage of sensitivity for culture from the CDPN model is higher than the nominal 95% levels for both the CDN and CDPN data generating mechanisms with the upper limits of the confidence intervals approaching 100%.

**Table 3:** 95% Coverage probabilities and 95% confidence intervals for sensitivity estimates across 1000 simulations

		Culture				
Model		GS Model	CIndep Model	CDP Model	CDN Model	CDPN Model^a^
Data generating mechanism	CIndep	**0(0–0.3)**	96.4(95.1–97.4)	97.4(96.3–98.3)	97.0(95.8–97.9)	96.8(95.6–97.8)
	CDP	**0(0–0.3)**	94.7(93.2–96.0)	94.8(93.3–96.0)	**93.5(91.8–94.9)**	96.6(95.3–97.6)
	CDN	**0(0–0.3)**	**81.0(78.5–83.3)**	**79.1(76.5–81.5)**	95.5(94.1–96.7)	99.2(98.5–99.6)
	CDPN	**0(0–0.3)**	**92.2(90.4–93.7)**	**84.2(81.8–86.4)**	96.7(95.5–97.7)	99.5(98.9–99.8)
		Serology				
Model		GS Model	CIndep Model	CDP Model	CDN Model	CDPN Model^a^
Data generating mechanism	CIndep	96.2(95.5–96.7)	97.0(96.4–97.4)	96.8(96.3–97.3)	96.6(96.0–97.1)	96.8(96.2–97.3)
	CDP	**89.1(88.1–90.0)**	**80.4(79.1–81.6)**	95.5(94.8–96.1)	**79.4(78.1–80.6)**	95.0(94.3–95.6)
	CDN	95.4(94.7–96.0)	96.5(95.9–97.1)	96.5(95.8–97.0)	96.9(96.3–97.4)	97.2(96.6–97.7)
	CDPN	**88.4(87.4–89.4)**	**87.5(86.5–88.5)**	97.4(96.8–97.8)	**85.2(84.1–86.3)**	97.0(96.4–97.5)

Values in bold show scenarios where the upper limit of the confidence interval is less than 95%. Confidence intervals for coverage calculated using Jeffreys prior

^a^Total number of simulations summarised is not equal to 1000 for the CDPN model due to a number of simulations with convergence problems

**Table 4:** 95% Coverage probabilities and 95% confidence intervals for specificity estimates across 1000 simulations

		Culture				
Model		GS Model	CIndep Model	CDP Model	CDN Model	CDPN Model^a^
Data generating mechanism	CIndep	**0(0–0.3)**	99.8(99.4–100)	100(99.7–100)	99.8(99.4–100)	99.9(99.5–100)
	CDP	**0(0–0.3)**	**8.3(6.7–10.1)**	96.9(95.7–97.8)	**8.4(6.8–10.2)**	96.7(95.5–97.7)
	CDN	**0(0–0.3)**	99.0(98.2–99.5)	100(99.7–100)	98.7(97.9–99.3)	99.9(99.5–100)
	CDPN	**0(0–0.3)**	**12.5(10.6–14.7)**	97.8(96.7–98.6)	**12.1(10.2–14.2)**	97.3(96.2–98.2)
		Serology				
Model		GS Model	CIndep Model	CDP Model	CDN Model	CDPN Model^a^
Data generating mechanism	CIndep	**0(0–0.1)**	96.1(95.5–96.7)	96.0(95.4–96.6)	96.5(95.9–97.1)	97.5(96.9–97.9)
	CDP	**0(0–0.1)**	95.4(94.7–96.0)	97.8(97.3–98.2)	96.2(95.6–96.8)	98.4(98.0–98.7)
	CDN	**0(0–0.1)**	97.5(96.9–97.9)	97.6(97.1–98.0)	97.5(97.0–98.0)	97.6(97.1–98.0)
	CDPN	**0(0–0.1)**	94.7(93.9–95.3)	97.3(96.8–97.8)	95.3(94.6–95.9)	98.1(97.6–98.5)

Values in bold show scenarios where the upper limit of the confidence interval is less than 95%. Confidence intervals for coverage calculated using Jeffreys prior

^a^Total number of simulations summarised is not equal to 1000 for the CDPN model due to a number of simulations with convergence problems

Specificity estimates for all models, except the GS model, show good coverage for serology tests (*j* = 2, .., 5). For culture (*j* = 1), coverage is higher than the nominal 95% level for all models except the GS model under CIndep and CDN data generating mechanisms. In the CDP and CDPN data generating mechanisms there is good coverage with models that account for the conditional dependence of tests in disease positive individuals (CDP and CDPN models) and poor coverage (< 15%) with models that do not account for conditional dependence of tests in disease positive individuals (GS, CIndep and CDN models).

### Precision

A complete table of precision estimates for each estimand within each scenario and for each model can be found in Supplementary Tables 2 and 3. Precision of estimates of sensitivity and specificity across all data generating mechanisms and models were similar for serological tests (*j* = 2, .., 5) but differed for our independent culture test (*j* = 1). For culture, the empirical standard error of estimates for both estimands was larger using the most general model, CDPN model, similar across CIndep, CDP and CDN models, and 0 for the GS model which assumes the test was perfect. The loss of efficiency from using the most general model (CDPN model) was high for estimating the accuracy of culture but low for estimating the accuracy of serological tests. However, if we just consider using the CDP model (accounting for conditional dependence in disease positive individuals only) the loss of efficiency from using this model when the true data generating mechanism is CIndep was never more than 2% for either estimand and all tests.

### Secondary simulation

**Fig. 2:**
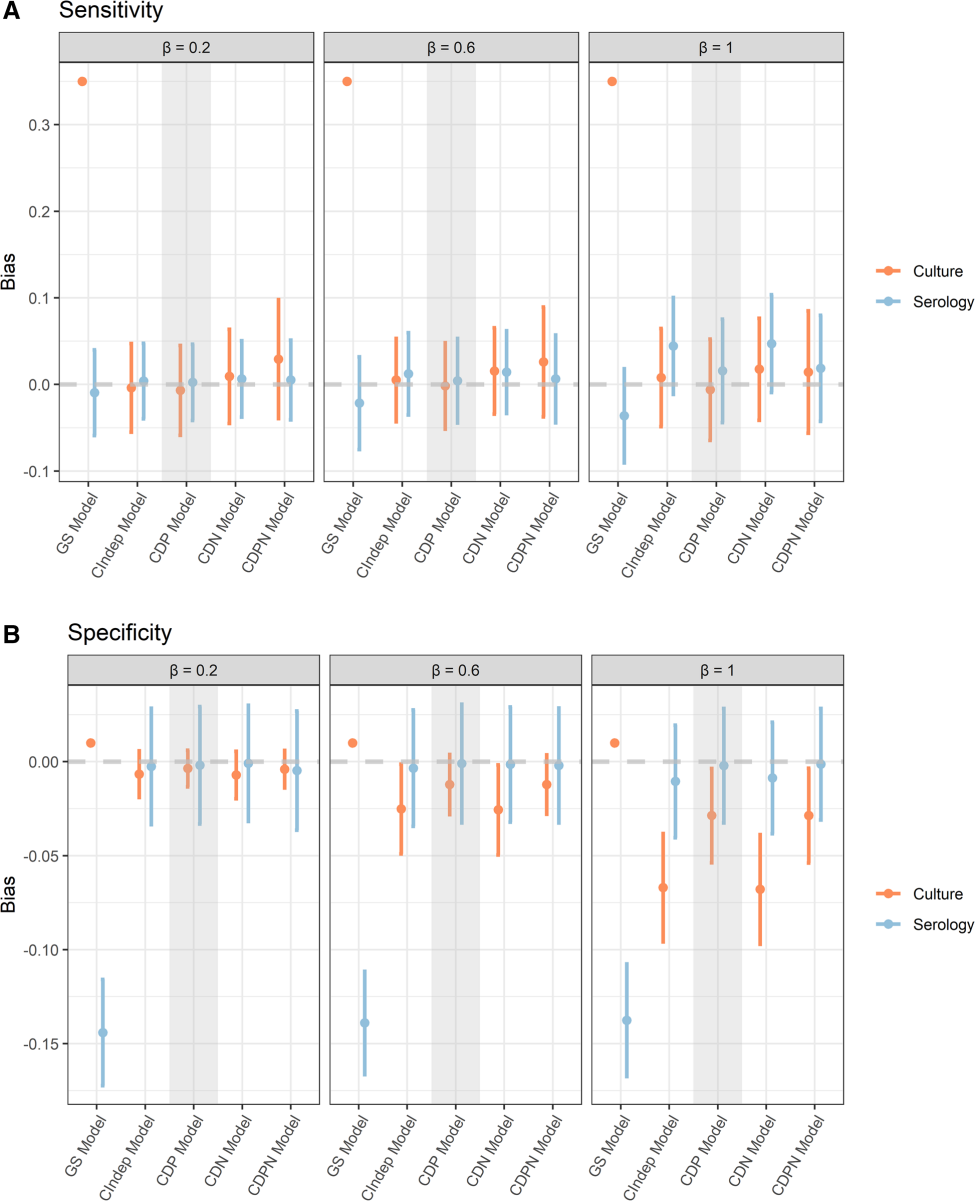
Mean bias and 95% confidence interval in estimates of sensitivity and specificity with varying *β* ’s. Points show mean bias across all valid simulations and the bar extends to the lower and upper confidence intervals. Shaded areas highlight the model that corresponds to the data generating mechanism

All results so far considered the scenario where the standard deviation for the random effect is equal to one. We also considered, in a secondary simulation, the bias in estimates of sensitivity and specificity at two other levels of the standard deviation for the scenario where there exists conditional dependence in serological tests among infected individuals (CDP). These results are shown in Fig. 2 and show that the size of bias increases as the value for the standard deviation increases when there exists conditional dependence but the model used assumes conditional independence among disease positive individuals. Among models where culture is assumed imperfect, this bias results in increasingly underestimated specificity estimates for culture.

### Convergence

All simulations in GS, CIndep, CDP and CDN models passed our convergence checks and had a reported rank normalised split- $\hat{R}$ statistic as < 1.01 and had no divergent transitions. The CDPN model reported convergence warnings in a number of simulations. 13% of simulations from the CDPN model under the CIndep data generating mechanism were removed along with 9, 3 and 10% in CDP, CDN and CDPN data generating mechanisms respectively. To run the CDPN and ensure there are no warnings, additional prior information may be required. In this analysis, all simulations with divergent transitions or a split- $\hat{R}$ statistic greater than 1.01 were removed from result summaries.

### Motivating example

We re-analysed the data used by Limmathurotsakul et al. [16] and extended their work by considering a dependence structure not considered in the original paper. We fitted Models 0–5 as defined in Table 1 and as considered in the simulation study. Estimates of the sensitivity and specificity under each model are presented in Table 5. The point estimates and width of 95% credible intervals are similar across models 0–3 (model 0 being the model assuming conditional independence and models 1 and 2 being models that account for conditional dependence between two tests using fixed effects) however models 3, 4 and 5 (random effect models) do exhibit some important differences. Between models 4 and 5 the median sensitivity of culture and specificity of ELISA differs by 7% while between models 3 and 5 the median specificity of serological tests differs by 2–9%.

**Table 5:** Sensitivity and specificity (95% credible interval) estimated from each model for each diagnostic test

Model Name | Assumed dependence structure		Model 0 CIndep	Model 1 CDP among two tests	Model 2 CDP among two tests	Model 3 CDP	Model 4 CDN	Model 5 CDPN
Effect Type Used		NA	Fixed	Fixed	Random	Random	Random
Test	Measure						
**Culture**	Se^a^	61(53–69)	62(54–69)	62(54–69)	60(52–69)	74(59–97)	67(57–79)
	Sp^b^	100^c^	100^c^	100^c^	100^c^	100^c^	100^c^
**Serology: IHA**	Se^a^	73(66–80)	73(66–79)	73(67–78)	70(63–76)	72(65–79)	69(62–75)
	Sp^b^	87(79–93)	86(79–93)	86(79–92)	84(75–92)	75(61–88)	76(66–84)
**Serology: Igm ICP**	Se^a^	81(75–86)	80(74–85)	80(74–86)	77(71–83)	80(72–86)	76(69–82)
	Sp^b^	65(56–74)	64(55–74)	65(56–73)	62(53–72)	56(45–67)	55(46–64)
**Serology: IgG ICT**	Se^a^	91(86–95)	91(86–94)	90(86–94)	88(82–92)	89(84–94)	87(81–92)
	Sp^b^	76(67–85)	75(66–84)	75(66–84)	74(64–85)	62(48–77)	65(54–74)
**Serology: ELISA**	Se^a^	77(70–84)	78(70–84)	78(71–84)	75(68–78)	82(74–88)	80(72–86)
	Sp^b^	97(93–100)	98(94–100)	97(93–100)	97(92–100)	88(72–99)	95(82–100)

Values shown are mean estimates with 95% credible intervals

^a^Se = sensitivity, ^b^Sp = specificity, ^c^Specificity assumed perfect. Models 0–4 were considered in the original work of Limmathurotsakul et al. while Model 5 is the additional analysis considered in this paper

Table 6 shows the expected frequency of each possible response profile from the 5 tests under each model. Viewing the results in this way as opposed to looking at just estimates of sensitivity and specificity highlights a few key things. It highlights the importance of allowing conditional dependence as model 0 (assuming conditional independence) appears to fit the data least well, and also shows that the structure of the conditional dependence modelled affects the fit. We can see that models which only consider dependence between two of the four serology tests (models 1 and 2), underestimate the frequency of extreme response profiles (i.e. 0,0,0,0,0 and 1,1,1,1,1). Model 3 accounting for conditional dependence between all serological tests in those disease positive is able to capture those with all positive response profiles but unsurprisingly fails to capture those will all negative response profiles. On the other hand, Model 4 exhibits the same tendencies in reverse while our additional model accounting for conditional dependence in both disease positive and disease negative individuals (Model 5) is able to capture both extremes and appears to fit the data best. This is confirmed by comparing the models on the expected log predictive density [24] where Model 5 shows the best predictive performance closely followed by Models 3 and 4 (See Supplementary Table 4 for more details).

**Table 6:** Observed and predicted frequency of each response profile from each model

		Expected frequency					
Response profile	Observed frequency	Model 0	Model 1	Model 2	Model 3	Model 4	Model 5
11111	69	49	53	53	63	49	65
11110	6	15	15	15	7	11	4
11101	0	5	6	1	2	6	2
11100	0	1	2	0	1	1	1
11011	9	12	8	13	6	13	7
11010	0	4	2	4	3	3	2
11001	0	1	1	0	1	2	1
11000	1	0	0	0	1	0	1
10111	14	18	14	14	11	19	12
10110	3	5	4	4	5	4	4
10101	0	2	1	6	1	3	1
10100	5	1	0	2	2	1	2
10011	3	4	9	3	4	5	6
10010	0	1	3	1	5	1	4
10001	3	0	1	2	1	1	2
10000	6	0	0	0	6	0	6
01111	35	31	33	33	42	31	35
01110	15	11	11	11	7	18	15
01101	0	3	4	1	1	3	2
01100	5	5	6	5	6	6	8
01011	5	8	5	8	4	4	4
01010	6	5	4	5	5	5	5
01001	0	1	1	0	1	1	1
01000	7	8	9	8	9	5	7
00111	5	12	9	9	8	9	7
00110	18	12	12	12	13	17	17
00101	0	2	2	5	2	2	1
00100	25	29	29	30	29	19	22
00011	7	3	6	3	3	3	3
00010	11	17	19	18	20	14	15
00001	2	1	2	2	2	2	2
00000	60	55	52	52	50	62	58

Observed frequency shown corresponds to five diagnostic test results from 320 patients with suspected melioidosis analysed in Limmathurotsakul et al. Models 0–4 were considered in the original analyses but model 5 is new to this paper

## Discussion

We carried out a simulation study investigating the bias and coverage of sensitivity and specificity estimates arising from mis-specification of the conditional dependence structure in latent class models. We found that assuming conditional independence among tests within disease positive or disease negative individuals when conditional dependence exists leads to bias and poor coverage in estimates of test accuracy. Due to the minimal loss in precision seen by using a model which accounts for conditional dependence between serology type tests in disease positive individuals, our results suggest it makes sense to account for conditional dependence in positive individuals even if researchers are unsure of its presence or if it is only expected at minimal levels. And, if there is a suggestion that there is dependence in both disease positive and disease negative individuals we would recommend using the most general model, particularly if the specificity of diagnostic tests being investigated are less than perfect. The results from this simulation also reiterate findings from previous studies [5, 14, 16] that assuming conditional independence between imperfect tests is still much better than assuming an imperfect test is a gold-standard, even when the conditional independence assumption is not valid.

Our simulation study revealed that the size of bias in estimates of sensitivity and specificity was greatest when there existed conditional dependence among disease positive individuals and latent class models used assumed conditional independence among disease positive individuals. The size of this bias increased as the standard deviation of the random effects increased. Bias was larger when conditional dependence existed among disease positive individuals than conditional dependence in disease negative individuals. This reflects the fact that the true specificity was reasonably high in our simulation at 90% compared to a moderate sensitivity of 65%. In similar scenarios, where specificity is generally believed to be higher than sensitivity, these findings highlight that considering dependence among the disease positive individuals is most important to reduce the bias in accuracy estimates.

In the motivating example, accounting for conditional dependence in only disease positive or disease negative individuals may have resulted in biased estimates of the sensitivity and specificity of tests included in this analysis. Comparing the model that only considered dependence in positive individuals and the model that considered dependence in both positive and negative individuals, the median specificity of one test differed by 9 percentage points. Although dependence among disease negative individuals was thought to be negligible, examining the predicted frequencies for each profile highlighted shortfalls in the final selected model which assumed independence among tests in these individuals. This was confirmed with a relatively novel model comparison tool that addresses shortfalls of earlier estimates such as AIC and DIC [24]. This re-analysis highlights that examining predicted frequencies, when you have a truth to compare to, might be a useful addition in investigating the most appropriate conditional dependence structure for a dataset.

There are limitations to this simulation study. Practically, we saw a limitation in implementing the CDPN model where some simulations exhibited divergent transitions and others a split $\hat{R}$ statistic greater than 1.01. In this case additional prior information may be necessary to ensure the model converges to the correct target distribution. Another limitation to this study is that we only considered a single prior distribution for the standard deviation of the random effect however estimates could be altered by a different choice of prior which has been investigated in a simulation study by Lee et al. [25]. We considered a single correlation among all serological tests in either disease positive or disease negative individuals. In practice you may have pairs or groups of tests that each require different random effect parameters with different standard deviations. However, if this is the case, this simulation still serves to highlight the potential biases that could be present in estimates of sensitivity and specificity if incorrect assumptions are made about the conditional dependence structure. Lastly, a key assumption of this simulation and our motivating example is that in the underlying population there exist only two disease classes; diseased and disease free. In some situations more than two classes may exist in a population, for example to distinguish between symptomatic and asymptomatic individuals. In cases where more than two diseases classes exist, recent work has shown that estimates of sensitivity and specificity from the two state LCM can be biased [26].

## Conclusions

The impact of biased estimates of sensitivity and specificity is twofold. Firstly, a test whose accuracy is underestimated may not be used when it could be useful (more accurate, cheaper or easier to implement) and secondly, a test whose accuracy is overestimated may be used when more useful tests exist. Both outcomes ultimately result in negative consequences for individuals and societies, so minimizing the bias in our estimates of diagnostic test accuracy is paramount. This paper serves to highlight that not only should conditional dependence be taken account of but that the choice of conditional dependence structure is important and should be considered in any analysis of diagnostic test accuracy that utilizes latent class models.

## Supplementary Information

**Additional file 1.**

**Additional file 2.**

**Additional file 3.**

**Additional file 4.**

## Data Availability

The dataset analysed during the current study are available from the supplementary material of the original article [16]. All code used in the current study to generate the simulated data sets and run each model are available from github (https://github.com/shk313/Evaluating-sensitivity-and-specificity-from-LCM-a-simulation-study.git).

## Abbreviations

LCM: Latent Class Model
AIC: Akaike Information Criterion
DIC: Deviance Information Criterion
