# Supplementary material for: Estimating sensitivity and specificity of diagnostic tests using latent class models that account for conditional dependence between tests: a simulation study
Source: BMC Med Res Methodol. 2023 Mar 10;23:58. doi: 10.1186/s12874-023-01873-0 (PMC9999546; doi:10.1186/s12874-023-01873-0)
Supplement: Supplementary file 1 — Additional file 1. [file 12874_2023_1873_MOESM1_ESM.docx]

Supplementary Table 1. Performance measures: definitions, estimates and Montel Carlo standard errors where these are considered

| Performance measure | Definition | Estimate | Monte Carlo standard error of estimate |
| --- | --- | --- | --- |
| Bias | $E\left[ \hat{\theta} \right]-\theta$ | $\frac{1}{n_{i}}\sum_{i=1}^{n_{i}} \hat{\theta_{i}}-\theta$ | $\sqrt{\frac{1}{n_{sim}\left( n_{sim}-1 \right)}\sum_{i=1}^{n_{sim}} (\hat{\theta_{i}}}-{\bar{\theta})}^{2}$ |
| Empirical standard error | $\sqrt{Var\left( \hat{\theta} \right)}$ | $\sqrt{\frac{1}{n_{i}-1}\sum_{i=1}^{n_{i}} (\hat{\theta_{i}}}-{\theta)}^{2}$ |  |
| Coverage | $Pr\left( \hat{\theta_{low}}\leq\theta\leq\hat{\theta_{upp}} \right)$ | $\frac{1}{n_{i}}\sum_{i=1}^{n_{i}} 1\left( \hat{\theta_{low,i}}\leq\theta\leq\hat{\theta_{upp,i}} \right)$ |  |

$\theta$ represents the true value of an estimand, $\hat{\theta}$ the estimator, $\hat{\theta_{i}}$ the estimate from the ith simulation, $\hat{\theta_{low}}$ and $\hat{\theta_{upp}}$ the estimate of the lower and upper 95% credible interval for the estimand respectively, $\bar{\theta}$ the mean of $\hat{\theta_{i}}$ across simulations and $n$ the number of simulations.$n_{sim}$ is the number of simulations considered and $i=1,\ldots,n_{sim},$ indexes a specific repetition of the simulations.
