# Supplementary material for: Estimating sensitivity and specificity of diagnostic tests using latent class models that account for conditional dependence between tests: a simulation study
Source: BMC Med Res Methodol. 2023 Mar 10;23:58. doi: 10.1186/s12874-023-01873-0 (PMC9999546; doi:10.1186/s12874-023-01873-0)
Supplement: Supplementary file 2 — Additional file 2. [file 12874_2023_1873_MOESM2_ESM.docx]

Supplementary Table 2: Empirical standard error of sensitivity estimates for each test type

|  |  | Serology $(j=2,..,5)$ | | | | |
| --- | --- | --- | --- | --- | --- | --- |
| Model |  | GS Model | CIndep Model | CDP Model | CDN Model | CDPN Model |
| Data generating mechanism | CIndep | 0.037 | 0.033 | 0.033 | 0.033 | 0.035 |
|  | CDP | 0.038 | 0.038 | 0.040 | 0.039 | 0.041 |
|  | CDN | 0.037 | 0.033 | 0.033 | 0.033 | 0.036 |
|  | CDPN | 0.038 | 0.038 | 0.039 | 0.039 | 0.041 |
|  |  | Culture $(j=1)$ | | | | |
| Model |  | GS Model | CIndep Model | CDP Model | CDN Model | CDPN Model |
| Data generating mechanism | CIndep | 0.000 | 0.036 | 0.036 | 0.037 | 0.155 |
|  | CDP | 0.000 | 0.040 | 0.041 | 0.041 | 0.047 |
|  | CDN | 0.000 | 0.036 | 0.036 | 0.039 | 0.057 |
|  | CDPN | 0.000 | 0.037 | 0.038 | 0.039 | 0.056 |
