# Supplementary material for: Estimating sensitivity and specificity of diagnostic tests using latent class models that account for conditional dependence between tests: a simulation study
Source: BMC Med Res Methodol. 2023 Mar 10;23:58. doi: 10.1186/s12874-023-01873-0 (PMC9999546; doi:10.1186/s12874-023-01873-0)
Supplement: Supplementary file 3 — Additional file 3. [file 12874_2023_1873_MOESM3_ESM.docx]

Supplementary Table 3: Empirical standard error of specificity estimates for each test type

|  |  | Serology $(j=2,..,5)$ | | | | |
| --- | --- | --- | --- | --- | --- | --- |
| Model |  | GS Model | CIndep Model | CDP Model | CDN Model | CDPN Model |
| Data generating mechanism | CIndep | 0.022 | 0.024 | 0.024 | 0.023 | 0.125 |
|  | CDP | 0.021 | 0.022 | 0.022 | 0.022 | 0.120 |
|  | CDN | 0.022 | 0.025 | 0.025 | 0.024 | 0.124 |
|  | CDPN | 0.021 | 0.025 | 0.025 | 0.024 | 0.119 |
|  |  | Culture $(j=1)$ | | | | |
| Model |  | GS Model | CIndep Model | CDP Model | CDN Model | CDPN Model |
| Data generating mechanism | CIndep | 0.000 | 0.008 | 0.006 | 0.008 | 0.048 |
|  | CDP | 0.000 | 0.021 | 0.017 | 0.021 | 0.152 |
|  | CDN | 0.000 | 0.009 | 0.007 | 0.009 | 0.167 |
|  | CDPN | 0.000 | 0.022 | 0.017 | 0.023 | 0.160 |
