# Supplementary material for: Estimating sensitivity and specificity of diagnostic tests using latent class models that account for conditional dependence between tests: a simulation study
Source: BMC Med Res Methodol. 2023 Mar 10;23:58. doi: 10.1186/s12874-023-01873-0 (PMC9999546; doi:10.1186/s12874-023-01873-0)
Supplement: Supplementary file 4 — Additional file 4. [file 12874_2023_1873_MOESM4_ESM.docx]

Supplementary Table 4: Motivating example model comparison on expected log predictive density

| Model | ELPD^a^ difference | SE^b^ difference |
| --- | --- | --- |
| Model 5 | 0.0 | 0.0 |
| Model 3 | -7.3 | 4.9 |
| Model 4 | -10.6 | 5.7 |
| Model 2 | -33.4 | 9.7 |
| Model 1 | -47.8 | 10.9 |
| Model 0 | -36670.0 | 19.1 |

^a^ Expected log predictive density difference is the difference in Bayesian leave one out estimate of the expected log pointwise predictive density between two models (elpd_loo). Comparison is made between each model and the model with the largest expected log predictive density. ^b^ Standard error of component-wide differences in elpd_loo.
